# Equivalent Fracture Patterns Demonstrate Poorer Postoperative Functional Outcomes Among Pronation-External Rotation IV Ankle Fractures

**DOI:** 10.7759/cureus.53348

**Published:** 2024-01-31

**Authors:** Wangsheng Wu, Bingsheng Liu, Chengwei Wang

**Affiliations:** 1 Orthopaedics, The Quzhou Affiliated Hospital of Wenzhou Medical University, Quzhou People's Hospital, Quzhou, CHN; 2 Orthopaedics, Affiliated Tumor Hospital of Xinjiang Medical University, Ürümqi, CHN

**Keywords:** ankle fracture, equivalent fracture, pronation external rotation, outcome, aofas

## Abstract

Background: Pronation-external rotation IV (PER IV) ankle fractures are relatively uncommon among rotational ankle fractures, but they are the most severe type. Although recent studies have shown satisfactory functional recovery in PER IV after surgical treatment, the different outcomes between fracture patterns and equivalent fracture patterns have not yet been evaluated. This study aims to compare short-term outcomes in PER IV ankle injuries between fracture patterns and equivalent fracture patterns.

Methods: This retrospective study was conducted at Quzhou Affiliated Hospital of Wenzhou Medical University, Quzhou, China, from July 2023 to October 2023. A total of 41 PER IV injuries from 2018 to 2022 were included and followed for at least one year. The American Orthopaedic Foot and Ankle Society (AOFAS) Ankle‑Hindfoot Scale, Ankle Range of Motions (ROM), and Visual Analogue Scale (VAS) for pain scores were the main outcome measures. The rate of postoperative complications was the secondary outcome measure. Patient demographics were compared in PER IV fractures and PER IV ankle equivalent fractures.

Results: The mean follow-up time was 18.2 ± 4.2 (range, 12-24) months. Postoperative X-ray and CT scans showed a satisfactory reduction of the ankle joint and syndesmosis. No reduction loss of distal tibiofibular syndesmosis or ankle joints was found at the 12-month follow-up. The average AOFAS scores after one year in both groups were satisfactory (fracture group vs. fracture equivalent group, 96.72 ± 4.21 vs. 92.63 ± 5.36; P < 0.05). The average VAS scores after one year in both groups were satisfactory (fracture group vs. fracture equivalent group, 1.45 ± 2.01 vs. 1.38 ± 1.96; P > 0.05). The average ROM scores after one year in both groups were satisfactory (dorsiflexion, fracture group vs. fracture equivalent group, 15.21 ± 5.62 vs. 13.46 ± 4.35; P > 0.05; plantar flexion, fracture group vs. fracture equivalent group, 38.62 ± 9.68 vs. 42.32 ± 5.28; P > 0.05).

Conclusion: For patients with PER-IV ankle injuries, the fracture mode had a better prognosis than the fracture equivalent mode.

## Introduction

Ankle fractures account for 10% of all fractures that occur throughout the body, and the incidence of ankle fractures in the lower extremity is second to hip fractures [1,2,3]. The two most widely used ankle fracture classification systems are the Lauge-Hansen and Weber-Danis classifications. The Lauge-Hansen classification is the most commonly used and consists of four types, namely, supination external rotation (SER), supination adduction (SA), pronation-external rotation (PER), and pronation abduction (PA) [4]. PER ankle fractures are relatively rare among these four types but are the most severe injuries and have been reported to account for 14-22% of ankle fractures [5-7]. According to the Lauge-Hansen classification, PER fractures can be further divided into four stages according to the extent of injury. Stage 1 is an avulsion fracture of the medial malleolus or rupture of the deltoid ligament, and stage 2 is a rupture or avulsion fracture of the anterior ligament of the distal tibiofibular syndesmosis. If the trauma continues and then progresses to stage 3, that is, a fracture at a relatively high position in the lateral malleolus, the fracture line is from anterosuperior to posteroinferior. If the trauma continues, it will progress to stage 4, which is a rupture or avulsion fracture of the posterior inferior tibiofibular ligament.

Clearly, a PER IV injury is associated with higher force exerted leading to injury mechanisms [8]. It can also be seen that there are two modes of PER IV injury, namely, fracture pattern and ligament rupture pattern (also known as the equivalent fracture pattern). The concurrent presence of medial malleolar fracture, distal tibiofibular syndesmotic anterior ligament avulsion fracture, lateral malleolar fracture, and distal tibiofibular syndesmotic posterior ligament avulsion fracture was defined as fracture patterns. The presence of one or more ligament ruptures other than avulsion fractures in the deltoid ligament or the anterior or posterior ligament of the distal tibiofibular syndesmosis was defined as a ligament rupture pattern.

As far as we know, only a few studies in the literature have compared the outcomes between these two modalities. The aim of this study was to compare the short-term outcomes of ankle PER IV fracture patterns with those of equivalent fracture patterns. We hypothesized that patients with ankle PER IV fracture patterns would have better functional outcomes than patients with equivalent fracture patterns.

## Materials and methods

The study was conducted at Quzhou Affiliated Hospital of Wenzhou Medical University, Quzhou, China, and approved by the Ethics Committee of the hospital, prior to a retrospective review of the medical record system. This retrospective study was conducted from July 2023 to October 2023. The study was conducted on all adult (>18 years of age) patients with PER IV ankle injuries from January 1, 2018 to January 1, 2022. The inclusion criteria are (1) patients aged 18-65 years old, (2) closed ankle fracture, (3) less than three weeks from injury to admission, and (4) PER type IV fracture according to the Lauge-Hansen classification. The exclusion criteria are (1) local skin infections before the injury, (2) ankle osteoarthritis before the injury, (3) history of an ankle trauma and surgery, (4) patients with severe osteoporosis, (5) ankle function being limited before the injury, (6) bilateral ankle fractures, (7) a Maisonneuve fracture, and (8) combined ipsilateral midfoot or hindfoot fractures (Table 1).

**Table 1:** Inclusion and exclusion criteria

Inclusion criteria	
	Aged 18-65 years
	Closed ankle fracture
	Less than 3 weeks from injury to admission
	Pronation-external rotation type IV fracture
Exclusion criteria	
	Local skin infection before injury
	Ankle osteoarthritis before injury
	History of ankle trauma and surgery
	Patients with severe osteoporosis
	Ankle function was limited before the injury
	Bilateral ankle fractures
	Maisonneuve fracture
	Combined ipsilateral midfoot or hindfoot fractures

A total of 41 patients were included in this study. The patients underwent manual reduction, with a short leg cast applied in the emergency room, and immediate fluoroscopy was performed to ensure that all patients were free of joint dislocation. Following admission, smokers were advised to abstain from smoking, while patients with comorbidities, such as diabetes and hypertension, were managed with the appropriate treatment. The affected limb was elevated to alleviate swelling, and a cold compress was applied locally. In addition, deep vein thrombosis was prevented through appropriate anticoagulant treatments. Surgery was possible when the swelling of the ankle subsided (skin wrinkles appeared). Preoperative X-ray and CT scans of the bilateral ankle joints were performed in all the patients.

All patients underwent open reduction and internal fixation under lumbar or general anesthesia. After the patient entered the operating room, the foot and ankle of the affected limb were immediately pre-sterilized by polyvinyl pyrrolidone (PVP). Surgery was performed in the floating position with the healthy limb straightened on a radiolucent operating table. All operations were performed with the assistance of an inflatable tourniquet. To prevent infection, cefuroxime or clindamycin was administered intravenously 30 minutes before surgery. All operations were performed by two surgeons on the same team.

Fractures of the fibula or medial malleolus were fixed according to standard Albeitgemeinshaft fur Osteosynthenfragen/Association for the Study of Internal Fixation (AO/ASIF) principles. The great saphenous vein and saphenous nerve should be protected. If the patient had an avulsion fracture of the medial malleolus, any fibers interposed were removed, and an arthrotomy into the ankle joint was performed allowing inspection of the medial articular surface. Then, the medial malleolus was reduced, and two cannulated screw guide pins were inserted. If the medial malleolar fragment is too small, a cannulated screw guide wire and one or two Kirschner wires are inserted. If the deltoid ligament ruptured, the deep or shallow deltoid ligament was repaired with one suture anchor separately. To repair the deep layer, one suture anchor (Smith & Nephew, USA) was inserted into the talus where the ligament was inserted. One suture was used to repair the deep layer of the deltoid ligament, and the other suture was used to pass through the bone tunnel of the interstitial sulci to strengthen the deep layer of the deltoid ligament. All the sutures were tightened without knotting.

To repair the shallow layer, one suture anchor (Smith & Nephew, USA) was inserted into the anterior colliculus, and the suture was used to repair the shallow layer. All the sutures were tightened without knotting. Attempting to reduce the fibula, if successful, Kirschner wires were used for temporary fixation. If anatomical reduction could not be obtained, temporary fixation was abandoned. Subsequently, the gap between the peroneal muscle and the hallus long flexor muscle was entered to expose the posterior malleolus, and then the hematoma and the embedded soft tissue were removed. The posterior malleolus was reduced and two cannulated screw guide pins were inserted under direct vision. Then, the fibula was reduced and temporarily fixed with Kirschner wires. Finally, the anterior syndesmosis ligament and its insertion were exposed through the lateral incision. If the ligament was ruptured, one suture anchor (Smith & Nephew, USA) was inserted to repair the ligament. If the patient had an avulsion fracture of the anterior syndesmosis ligament, the Wagstaffe or Chaput fragment was reduced and fixed with a cannulated screw guide pin. When the position was satisfactory after fluoroscopy, the lateral malleolus was fixed with a lateral malleolar anatomical plate or reconstruction plate. After reaming, 4.0 cannulated screws (Wego, China) were inserted with or without spacers, all guide pins were pulled out, and all reserved sutures were tightened and knotted. In the end, the Cotton test was performed, and all were negative. After irrigation, a negative-pressure drainage tube was placed in the posterior ankle, and then the wound was sutured and covered with a sterile dressing. The cold compress was bandaged from the metatarsophalangeal joint of the forefoot to the tibial tubercle below the knee.

Postoperatively, elevation of the affected limb, detumescence, analgesia, and appropriate anticoagulant treatment were continued to prevent deep vein thrombosis. The patient was instructed and assisted with functional exercises, including ankle plantar flexion and dorsal extension, knee flexion and extension, metatarsophalangeal flexion and extension, and quadriceps isometric contraction. On the second day after surgery, the wound dressing was changed, the drainage tube was removed, and the patient was instructed and assisted in performing functional exercises. After the surgical wound showed no signs of infection, the patients were typically discharged on the seventh postoperative day after surgical trauma evaluation. A follow-up examination was scheduled for all patients at the outpatient department at one, two, six, and 12 months after surgery. The patients were allowed to begin partial weight-bearing while walking after two months of follow-up.

X-ray and CT examinations were performed on the injured ankle on the second day after surgery, as shown in Figures 1-8.

**Figure 1:**
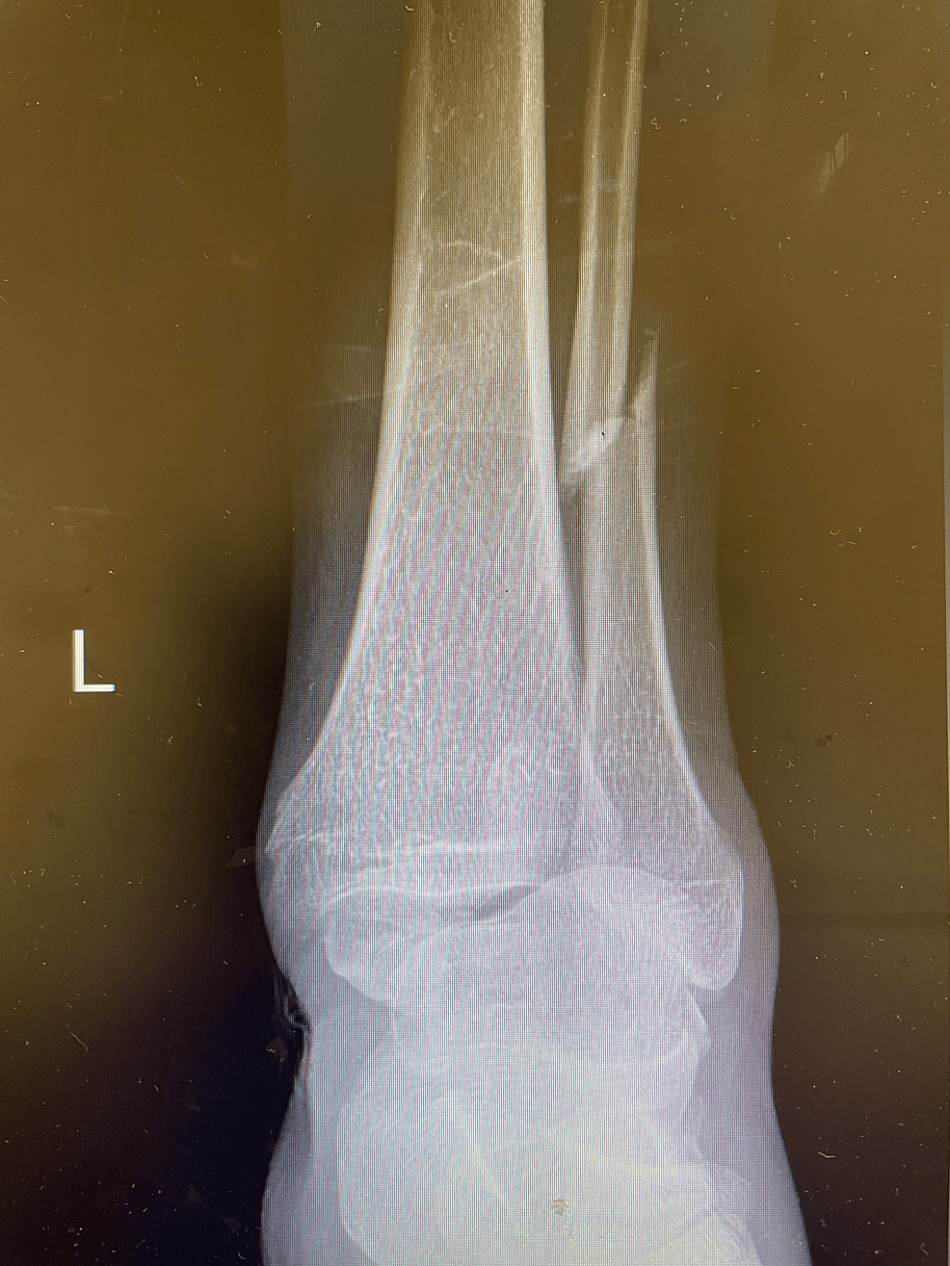
A 56-year-old male patient involved in a traffic accident with an ankle PER IV equivalent fracture with ankle radiograph. PER IV: pronation-external rotation IV

**Figure 2:**
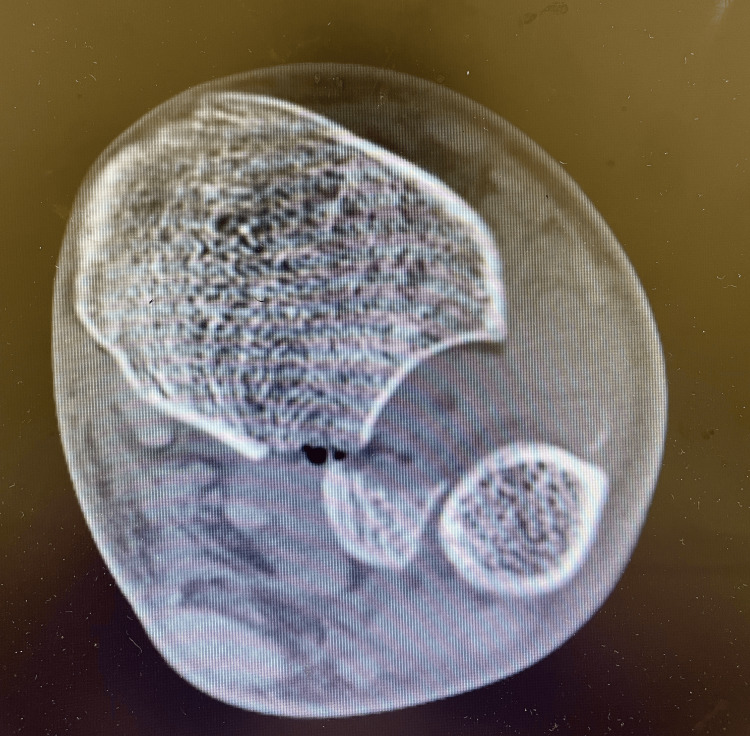
Axial CT scan of the ankle shows the posterior malleolar fracture and rupture of the anterior ligament syndesmosis.

**Figure 3:**
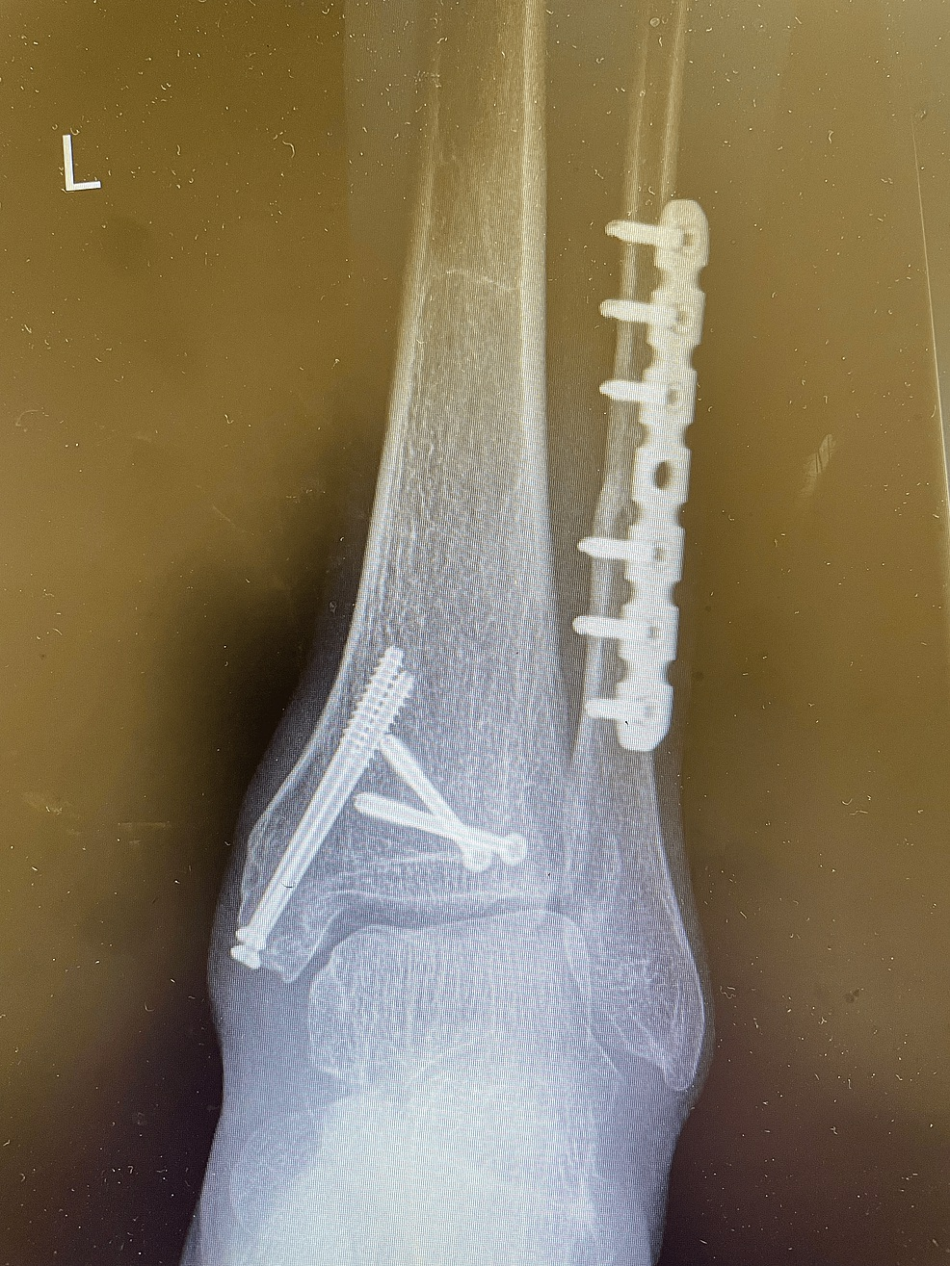
Postoperative anteroposterior radiograph. The lateral malleolus was fixed with reconstruction plates, the posterior malleolus and medial malleolus were fixed with cannulated screws, and the inferior tibiofibular anterior ligaments were repaired with suture anchors.

**Figure 4:**
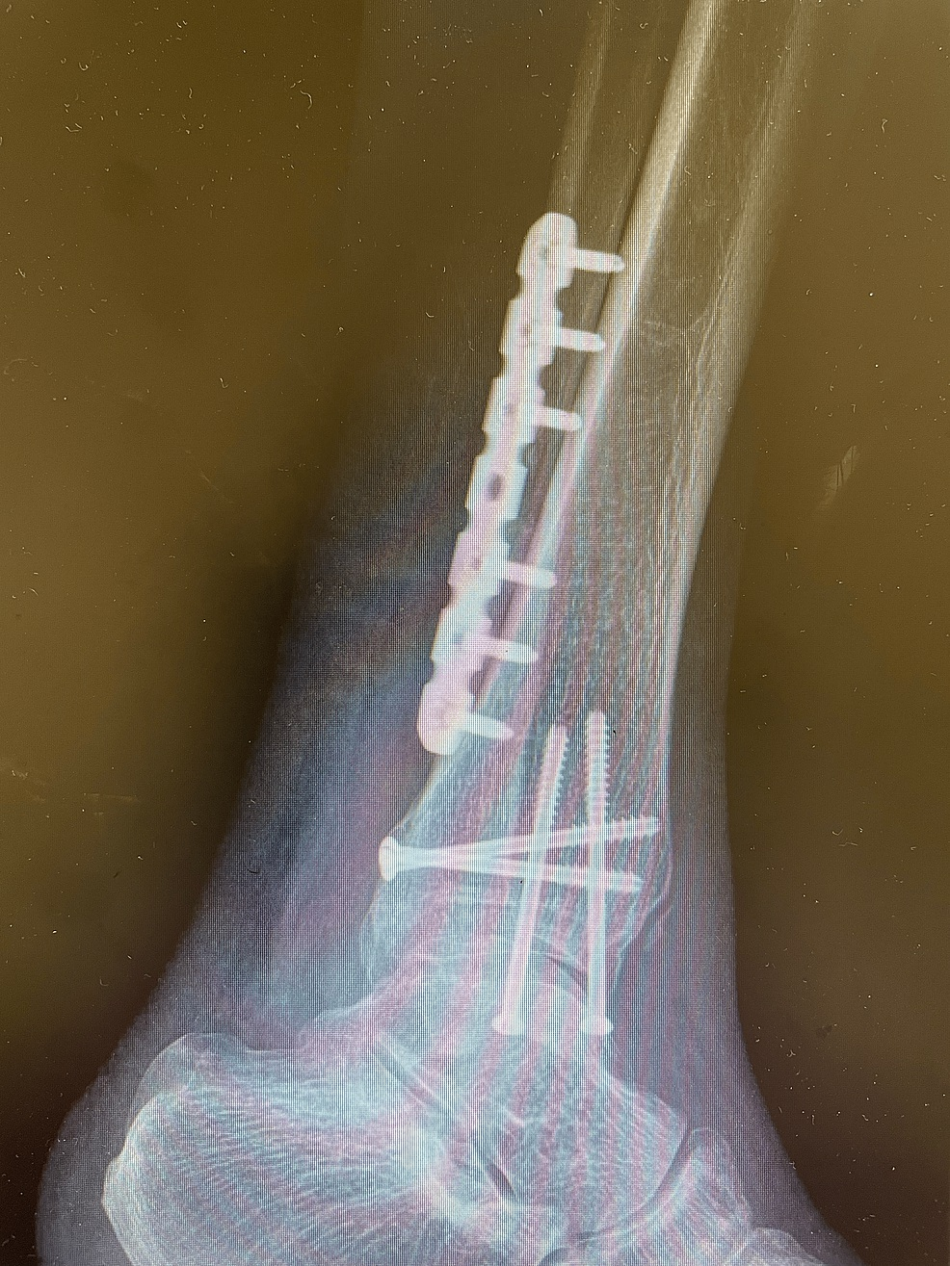
Lateral postoperative view.

**Figure 5:**
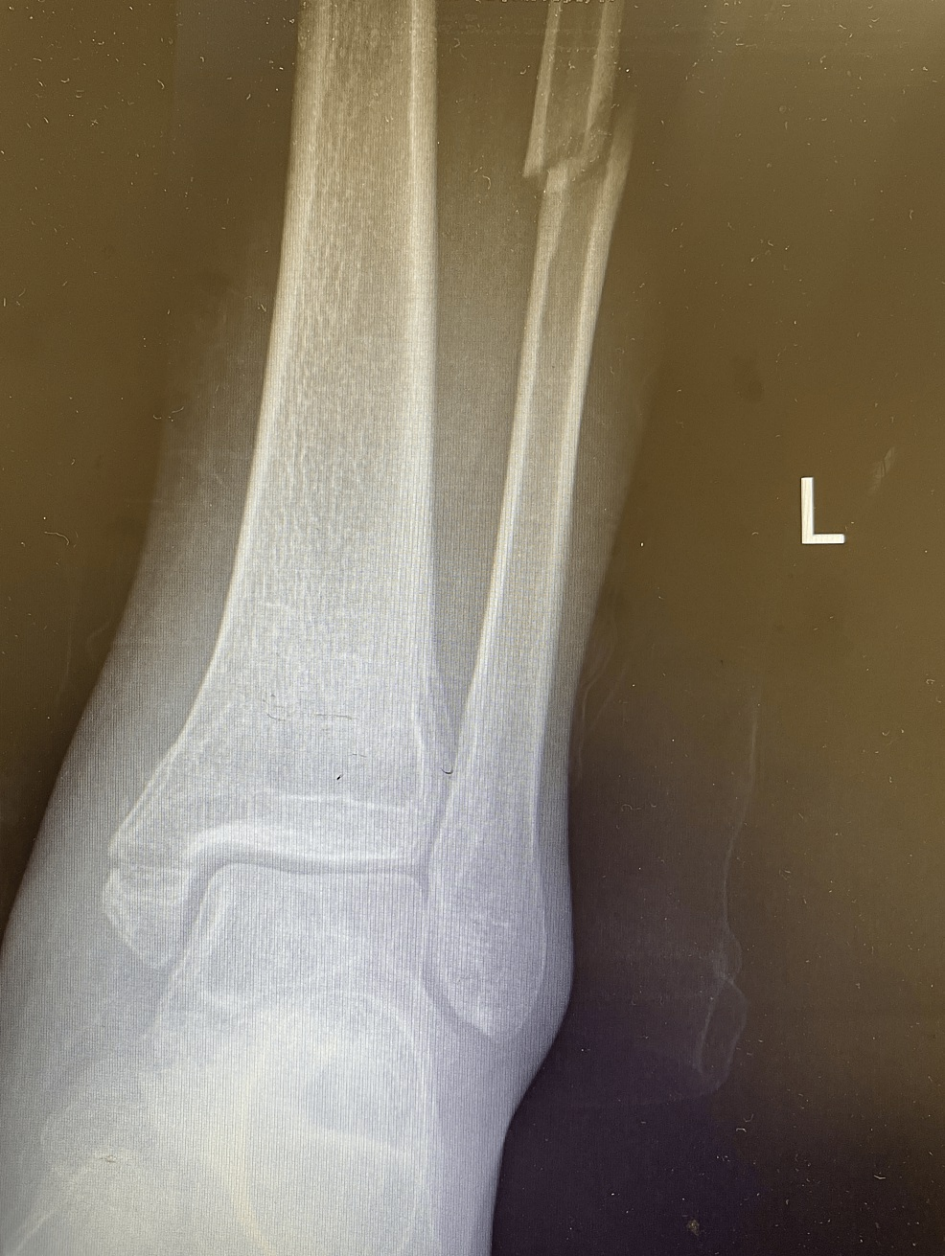
Anteroposterior ankle radiograph of a 68-year-old male presenting an ankle PER IV fracture.

**Figure 6:**
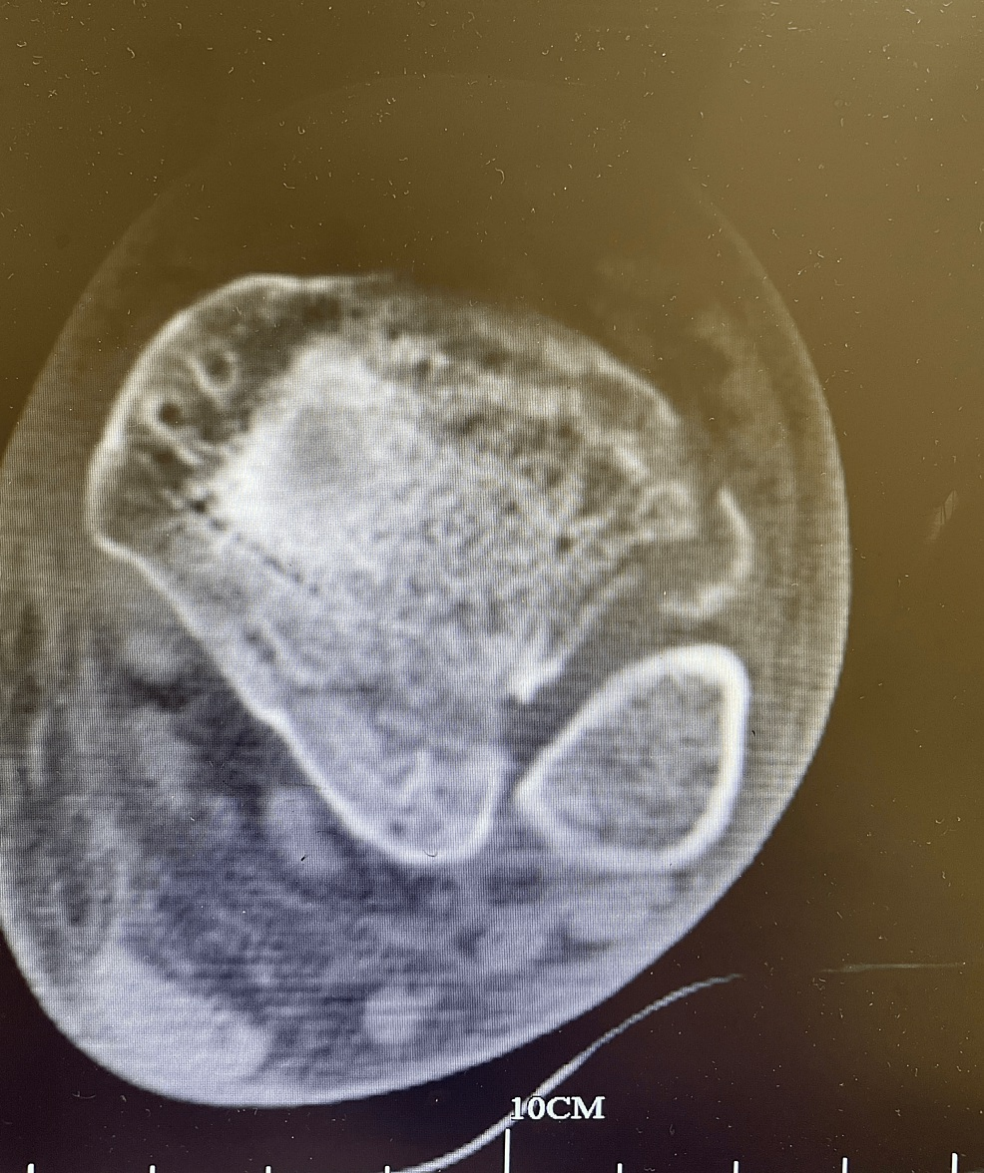
Axial CT scan of the ankle shows the posterior malleolar fracture and anterior malleolar fracture.

**Figure 7:**
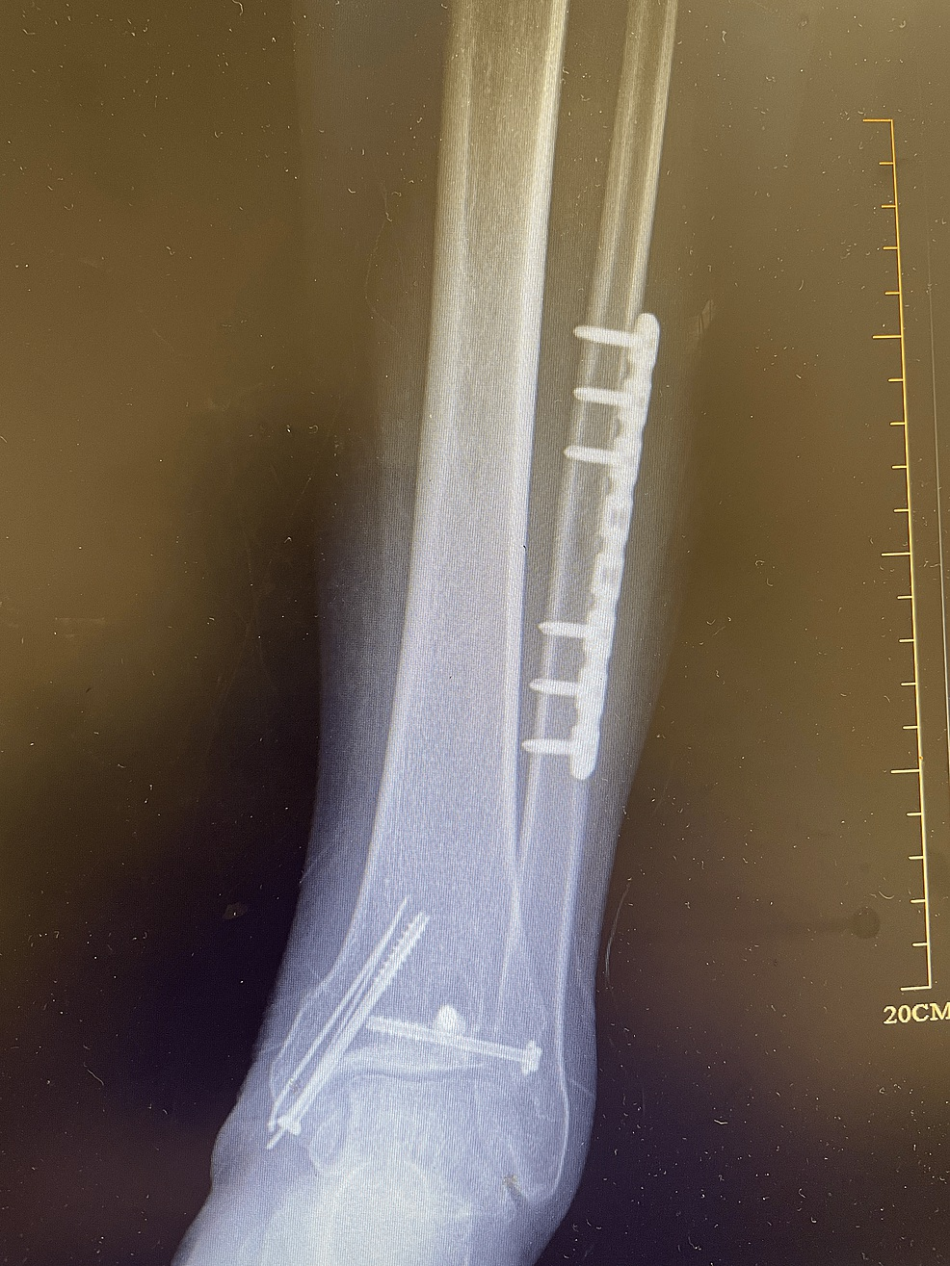
Postoperative anteroposterior radiograph. The lateral malleolus was fixed with reconstruction plates, and the posterior malleolus was fixed with a cannulated screw. Because the medial malleolar fragment is too small, it is fixed with a cannulated screw and three Kirschner wires.

**Figure 8:**
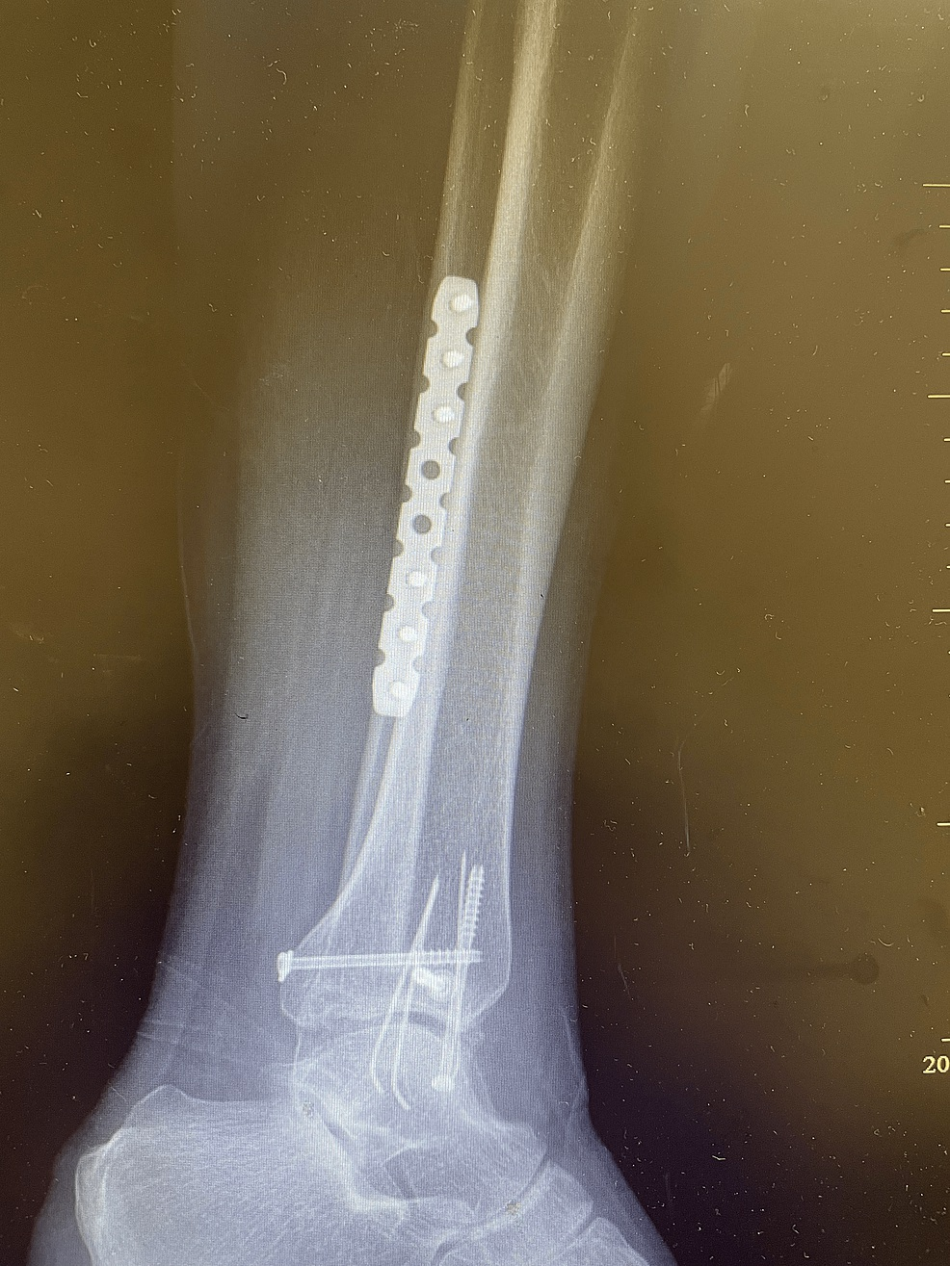
Lateral postoperative view shows anatomical reduction of fracture and satisfactory reduction of the ankle joint.

The primary clinical outcomes were assessed via the latest postoperative AOFAS, ankle range of motions (ROM), and visual analog scale (VAS). The rate of postoperative complications was the secondary outcome measure, including joint malreduction, distal tibiofibular syndesmosis malreduction, postoperative reduction loss, deep infection, and incision complications. Joint malreduction was defined if postoperative radiographic findings did not meet any of the following criteria: (1) trilateral intervals of the ankle joint should be equal and parallel, (2) the medial spike of the fibula should indicate the level of the tibial subchondral bone (no irregular Shenton’s line), (3) the contour of the lateral part of the articular surface of the talus continues as an unbroken curve to the recess in the distal fibula (Coin sign, and (4) no loose bodies in the joint [9-11]. Axial CT scan images were judged for the quality of reduction of the syndesmosis by measuring the distance between the fibula and the anterior and posterior facets of the incisura. Differences between the anterior and posterior measurements of more than 2 mm were considered malreduction [12]. A greater than 2-mm change in the medial clear space upon the comparison of the immediate postoperative and final radiographs was defined as a loss of reduction.

IBM SPSS Statistics for Windows, version 20 (released 2011; IBM Corp., Armonk, New York, United States) was used for analysis. Continuous variables were expressed as the mean ± standard deviation, and categorical variables were expressed as absolute values and percentages. Associations between categorical variables were tested by χ2 or Fisher exact tests. Relationships between continuous variables were tested by independent samples t-test, and non-normal distribution was tested by the Mann-Whitney U test. Statistical significance was defined as P < 0.05.

## Results

Data from 41 consecutive patients were included. Overall, the patients' age at the time of surgery was 34.5 ± 18.6 years (range 18-65 years). The fracture group consisted of 11 women and six men (17/41, 41%), and the fracture equivalent group consisted of eight women and 16 men (24/41, 59%). The patient demographic characteristics are shown in Table 2.

**Table 2:** Demographic data of the PER equivalent and PER fracture groups. PER: pronation-external rotation

Variables	Fracture group (n ＝ 17)	Fracture equivalent group (n＝24)	p-value
Age (years)	35.68±22.84	33.82±21.68	0.729
Gender			
Female	11	8	0.047
Male	6	16	
Dominant side			
Right	16	22	0.767
Left	1	2	
Affected side			
Dominant	10	15	0.812
Nondominant	7	9	

All patients were followed up for 12-24 months, with an average of 18 months. None of the patients developed an infection postoperatively. Eight patients presented mild signs of infection, such as swelling around the surgical wound. Three of 17 (18%) patients were in the fracture group, and 5/24 (21%) patients were in the fracture equivalent group. All of them healed after active symptomatic treatment (dressing change).

All 41 patients achieved bone healing six months after the operation. Postoperative X-ray and CT showed satisfactory reduction of the ankle joint and syndesmosis. No reduction loss of distal tibiofibular syndesmosis or ankle joints was found at the 12-month follow-up. The AOFAS score, VAS score, and ROM were recorded at 12 months after the operation. The average AOFAS scores after one year in both groups were satisfactory (fracture group vs. fracture equivalent group, 96.72 ± 4.21 vs. 92.63 ± 5.36; P < 0.05), although the value was higher in the fracture group, and the difference was statistically significant. The average VAS scores after one year in both groups were satisfactory (fracture group vs. fracture equivalent group, 1.45 ± 2.01 vs. 1.38 ± 1.96; P > 0.05). The average ROM scores after one year in both groups were satisfactory (dorsiflexion, fracture group vs. fracture equivalent group, 15.21 ± 5.62 vs. 13.46 ± 4.35; P > 0.05; plantar flexion, fracture group vs. fracture equivalent group, 38.62 ± 9.68 vs. 42.32 ± 5.28; P > 0.05). The primary outcomes are shown in Table 3.

**Table 3:** Comparison of ankle functions between the fracture group and fracture equivalent group AOFAS: American Orthopedic Foot and Ankle Society; VAS: visual analog scale; ROM: range of motion

Variables	Fracture group	Fracture equivalent group	p-value
AOFAS score	96.72 ± 4.21	92.63 ± 5.36	0.012
VAS score	1.45 ± 2.01	1.38 ± 1.96	0.912
ROM			
Dorsiflexion	15.21 ± 5.62	13.46 ± 4.35	0.268
Plantarflexion	38.62 ± 9.68	42.32 ± 5.28	0.123

## Discussion

In the present study, we compared the short-term functional outcomes of PER type IV ankle injuries in two modalities. We hypothesized that patients with PER IV injury in the fracture equivalent model would have worse functional outcomes than those with PER IV injury in the fracture model, and the results of this study support our hypothesis.

In the present study, patients with PER IV fractures had satisfactory functional recovery, and the results were also consistent with those of previous studies. Donken et al. reported that the long-term result of surgical treatment of PER ankle fractures is good or excellent in the majority of patients in a retrospective study with up to 20 years of follow-up [13]. Schottel et al. reported that fracture fragment and ligament-specific fixation resulted in good short-term outcomes in a cohort of operatively treated PER IV fractures [8]. In addition, the present study showed that the PER IV injury fracture type was more common in women, and the fracture equivalent type was more common in men, which is consistent with reports in the literature [4]. Casciato et al. reported that in PER IV injuries, the fracture group was mostly female, while the equivalent fracture group was more male, the difference was statistically significant, and the PER IV fracture equivalent group had significantly higher bone mineral density (BMD) than the fracture group [14]. A study of SER IV ankle injuries showed that the majority of patients in the equivalent fracture group were female, and the difference was statistically significant. The study also showed that the BMD was related to the type of injury, and patients with osteoporosis were more likely to suffer from fracture-equivalent model injury. It was suggested that physicians could predict bone quality based on an injury model to guide surgical strategies [15].

By contrast, the results of our PER IV equivalent fracture group differed from previously reported findings. In the present study, the postoperative function of the equivalent fracture group was worse than that of the fracture group, and the difference was statistically significant. Levack et al. studied postoperative functional recovery in different injury modes in SER IV injury and PER IV injury, and the results showed no difference in postoperative function between the fracture group and the equivalent fracture group [16].

In the present study, we treated PER IV injury with a total repair strategy, emphasizing the restoration of all injured components through fragment-specific fixation and repair of ruptured ligaments [17-22]. In the fracture group, the specific bone fragments were anatomically reduced and rigidly fixed. In the equivalent fracture group, we performed ligament repair for all ruptured ligaments. Theoretically, the ankle ring and the distal tibiofibular syndesmosis ligament ring were all intact after surgery. Satisfactory reduction of the ankle joint and a good relationship of distal tibiofibular syndesmosis were confirmed by imaging examination after the operation and during follow-up. All patients began functional exercise on postoperative day one.

It is well known that the functional recovery of ankle fractures mainly depends on anatomical reduction during surgery and the maintenance of anatomical reduction during postoperative functional exercise. In addition, functional exercise should be started as soon as possible after the operation [23,24]. Why did the difference occur in postoperative functional recovery between the two groups? We suspect that this is partly due to the pattern of injury. In the fracture group, the physiological function of the ligament was completely restored after fixation of the specific bone fragment. However, although ligament repair in the equivalent fracture group restored ligament continuity, the length of the ligament may not have reached the anatomical recovery, which was difficult to judge during the operation and can only be measured empirically by the operator. In addition, the ruptured ligament healed in the form of a scar after repair, and the scar ligament lost some of the elasticity that the ligament should have. Nakamura et al. found that the scar tissue formed by ligament healing has more water content, the normal large diameter type I collagen fibers are replaced by a large number of small type II collagen fibers, and type I collagen fibers are the main loading component of the ligament [25]. Therefore, the scar tissue formed after ligament healing is lower than that of the normal ligament in terms of mechanical properties. However, in the fractured group, anatomical reduction and rigid fixation were achieved to restore not only the continuity of the ligament but also the mechanical properties of the ligament.

A limitation of this study is the retrospective design. In the collection of postoperative evaluation indicators, due to inaccuracies in the medical records, the collected data may not be consistent with the actual data. In addition, although the operation was performed by two surgeons in the same group, the quality of the operation may be different at different times, although the patients in each group used similar reduction and fixation strategies. There is a need for future prospective studies.

## Conclusions

Through our study on PER-IV ankle injuries, we can conclude that patients with fracture patterns had a better postoperative function than those with equivalent fracture patterns. The difference between the two groups was found to be statistically significant. When managing PER IV injuries, orthopedic surgeons should inform patients in detail about the prognosis of different injury types, so that patients understand the different injury patterns and different functional recovery periods after operation to avoid medical disputes.

In the present study, all patients with PER IV injuries achieved good short-term outcomes. This further supports the concept of total reconstruction (fixing all four malleolar) regarding this injury pattern. We hope that these findings will help to improve the understanding of PER IV fractures.
